# Investigating the effect of synthesis selection on O3-sodium layered oxide structural changes and electrochemical properties

**DOI:** 10.3389/fchem.2023.1151656

**Published:** 2023-04-06

**Authors:** L. Acebo, N. E. Drewett, D. Saurel, F. Bonilla, T. Rojo, M. Galceran

**Affiliations:** ^1^ Center for Cooperative Research on Alternative Energies (CIC EnergiGUNE), Parque Tecnológico de Alava, Basque Research and Technology Alliance (BRTA), Vitoria-Gasteiz, Spain; ^2^ Departamento de Química Orgánica e Inorgánica, Universidad del País Vasco UPV/EHU, Bilbao, Spain

**Keywords:** sodium-ion batteries, layered oxide cathodes, synthesis, structural characterization, electrochemical characterization, insertion mechanism, phase change suppression

## Abstract

Transition metal (TM) layered oxides constitute a promising family of materials for use in Na-ion battery cathodes. Here O3-Na (Ni_1/3_Mn_1/3_Fe_1/3_) O_2_ was synthesised using optimised sol-gel and solid-state routes, and the physico- and electrochemical natures of the resulting materials were thoroughly studied. Significant differences in electrochemical behaviour were observed, and the use of in operando XRD determined this stemmed from the suppression of the P3 phase in the sol-gel material during cycling. This was attributable to differences in the degree of transition metal migration in the materials ensuing from the selection of synthetic route. This demonstrates that not only the choice of material, but also that of synthesis route, can have dramatic impact on the resulting structural and electrochemical nature, making such considerations critical in the future development of advanced Na-ion cathode materials.

## 1 Introduction

The dependence of modern society on the generation, storage, and distribution of electrical energy has led to these topics becoming areas of vital research—and, while a range of storage technologies exist, batteries remain critical to these efforts. Indeed, the ever-increasing demand on the energy infrastructure—from multiple sectors—is creating an impetus to develop application-led systems. In this way, devices may be developed with strengths which may be exploited and whose limitations are related to non-critical parameters. In short, it is imperative to develop a plethora of different battery technologies as a “toolbox” which may be tailored to meet specific application demands.

Sodium-ion (Na-ion) batteries are coming to prominence due to potential combination of attractive properties (such as low-cost, sustainability and flexible utility) and the relative abundance of their constituents (Slater et al., 2013; Bauer et al., 2018; Che et al., 2018)—particularly in comparison to analogous Lithium-ion (Li-ion) based systems (Palomares et al., 2013; Muñoz-Márquez et al., 2017; Vaalma et al., 2018). Nevertheless, despite this renaissance of Na-ion research, challenges remain facing these systems. Cathodes remain a key area for improvement, with a range of different systems available. These include polyanions (Senthilkumar et al., 2019; Berlanga et al., 2020; Chen et al., 2020), Prussian blue analogues (PBAs) (Li et al., 2019; Liu et al., 2020; Zhou et al., 2021), and Na-based transition metal layered oxides—with the latter proving particularly popular, as they are in many ways analogous to their lithium counterparts, which has led to rapid advances in this area (particularly as they may be produced *via* a scalable synthesis method and they can exhibit high theoretical capacities).

In brief, Na-based transition metal layered oxides (Na_x_MO_2_; where M represents one or more elements, typically including a transition metal) may be thought of as MO_2_ layers of edge-sharing MO_6_ octahedra with Na^+^ ions occupying interlayer space—which can be expressed as different structures depending on the oxygen stacking ordering (the notation for which was established by C. Delmas in the early 1980s) (Delmas et al., 1980; Yabuuchi et al., 2012; Han et al., 2015). As a general trend, the more ionic compounds adopt the O3 structure, while the more covalent prefer the O1 structure at high and low intercalant compositions (x ∼ 0 or x ∼ 1). At intermediate concentrations (x ∼ 1/2), however, the P3 structure is often preferred for intercalants that are large enough to support prismatic coordination (Radin and Van der Ven, 2016). Considerable work has been carried out investigating the O3 and P2 phases, which typically (though not exclusively) shows a strong link between the phase formed, the sodium content, and the resulting electrochemical performance—and though typically P2 phases are attractive due to their high working potential they are also sodium deficient, while by way of contrast the O3 phases are also of interest as their fully sodiated nature allows direct use in full cells (Pan et al., 2013; Fatima et al., 2021). Consequently, challenges remain—and one of the key ones is the large mechanical strain occurring during cycling, a result of the large Na-ion radius. This often leads to phase-transformations and structural rearrangements which—if not properly understood and handled—can result in poor structural stability and electrochemical performance (Wang et al., 2018).

Recent studies into the O3-Na (Ni_1/3_Mn_1/3_Fe_1/3_) O_2_ material (herein O3-NaNMF) have revealed interesting insights into the nature of the phase transitions during cycling (Xie et al., 2020) and electrochemical performance (Wang et al., 2016; Xie et al., 2016; Zhang et al., 2017; Sun et al., 2018; Xie et al., 2018; Jeong et al., 2019; Jung et al., 2020). In order to facilitate rapid, lab-scale materials development, we developed two novel, optimised synthetic routes to forming O3-Na (Ni_1/3_Mn_1/3_Fe_1/3_) O_2_. In this work we present both a one-pot, resorcinol-formaldehyde synthesis capable of forming nanoparticles (Han et al., 2005; Shaju and Bruce, 2008; Huang et al., 2010; Gao et al., 2015; Luo et al., 2016; Zheng et al., 2016; Guo et al., 2020), as well as a carbonate-precursor based solid state synthesis [the selection of precursors being carefully undertaken, resulting in a performance superior to that of the previously reported oxide-precursor solid-state synthesized O3-NaNMF (Zhang et al., 2017)]. In order to evaluate and compare these two facile synthetic routes, we present a comprehensive analysis of the structural, physicochemical and electrochemical properties of both materials, as well as offer a discussion of the observed differences. In this way, we offer insights into not only simple routes to validating sodium layered oxides, but also the important role the selection of synthetic route plays on the structural and electrochemical properties of the product.

## 2 Experimental

### 2.1 Solid state synthesis

Stoichiometric amounts of Na_2_CO_3_ (Sigma Aldrich, 99.5%), Fe (NO_3_)_3_ 9H_2_O (Sigma Aldrich, 99%), MnCO_3_ (Alfa Aesar 99.9%), and NiCO_3_ 2Ni(OH)_2_ (Fisher, 99%) were ball-milled with a sample mass: balls ratio of 20:1 at 250 rpm for 1 h. After, the powder was placed in a crucible at it was heated up to 920°C for 12 h under air atmosphere using a muffle (Carbolite, RHF 1600). Once the furnace was cooled down to ≈ 180°C, the pellet was quickly transferred to an Argon-filled glove box (<0.1 ppm H_2_O and O_2_) in order to minimize the exposure to air.

### 2.2 Sol-Gel Synthesis (hereafter O3-NaNMF-SG)

A stoichiometric ratio of resorcinol (Sigma Aldrich, 99%), NaNO_3_ (Sigma Aldrich, 99%), Ni (NO_3_)_2_ 6H_2_O (Sigma Aldrich, 99%), Mn (NO_3_)_2_ 4H_2_O (Sigma Aldrich, 98%) and Fe (NO_3_)_3_ 9H_2_O (Sigma Aldrich, 99%) was dissolved in water, and heated to 80°C. After reaching temperature, formaldehyde (37 wt%) was added and the solution stirred until polymerization of the resorcinol and formaldehyde initiated. Subsequently, this resorcinol-formaldehyde gel was cooled to room temperature, aged for 2 h, then dried at 90°C overnight (to evaporate the bulk of the water). Calcination was then carried out at 200°C for 2 h to form a powder, which was cooled to room temperature and reground. This powder was then heated at 5.5°C min^−1^–920°C for 12 h, then cooled to 180°C before transfer to a glove box under inert atmosphere (<0.1 ppm H_2_O and O_2_). The powder was then re-ground to give the final product.

### 2.3 X-Ray Diffraction (XRD)

The structural characterization was carried out using a Bruker D8 Discover X-ray diffractometer equipped with a LYNXEYE XE detector with Cu Kα radiation of *λ* = 1.54053 Å. The XRD patterns were refined using FullProf Suite program (Rodríguez-Carvajal, 1993). The *ex-situ* XRD patterns were remeasured by removing the electrodes form the used coin cell, which was firstly washed with dimethylcarbonate (DMC) and dried in order to remove the excess of the electrolyte.

### 2.4 Induced coupled plasma optical emission spectrometry (ICP-OES)

The amounts of sodium and transition metals were determined by using a Horiba Scientific Ultima 2 spectrometer (Jobin Yvon, Longjumeau, France) in conjunction with a AS500 autosampler and Activanalyst software (version 5.4). Digestion of materials was carried out using aqua regia (1:3 HNO_3_:HCl molar ratio) and stirring at room temperature for 3 days. A blank sample (containing aqua regia of the same concentration, treated under identical conditions) was also measured. Individual standard solutions of 1,000 mg L^−1^ of Fe, Mn, Na, and Ni supplied by Scharlab (Barcelona, Spain) was used for calibration. HNO_3_ 69% and HCl 37% from Scharlab (Barcelona, Spain) analytical grade and Ultrapure Water from Fischer Scientific (Waltham, Massachusetts, United States) were used. The analytical wavelengths used for the measurements are 216.556 nm Ni, 257.610 nm Mn, 259.940 nm Fe, and 588.995 nm Na. Concentrations of elements were quantified using four-point external calibration curves within the concentration range of [1–100] mg L^−1^. The intensities corresponding to the blank solution were subtracted from the intensities of the samples so as to obtain the final concentration of each element.

### 2.5 Electron microscopy

Morphology and homogeneity were studied by scanning electron microscopy (Thermofisher SEM-FEG Quanta 200) using a current accelerating voltage of 30 kV. Structural phase identification and compositional elemental analysis of the samples were carried out by transmission electron microscopy [Thermofisher TEM-FEG, Technai G2 F20 Super Twin (S-Twin)] operating at 200 kV by means the selected area electron diffraction (SAED) mode and the energy-dispersive X-ray spectroscopy (EDXS) respectively.

### 2.6 Electrochemical characterization

For electrochemical measurements, slurry formulation, the electrode preparation and cell assembling we carried out under argon atmosphere in a MBraun glove box (O_2_ and H_2_O ppm <0.1). O3-NaNMF powder, carbon C65, and poly (vinylidene fluoride) (PVdF; SOLEF) were mixed in a ratio of 80:10:10 with 1-methyl-2-pyrrolidone (NMP; Sigma Aldrich, 99.5%). The slurry was coated onto a battery-grade aluminium foil current collector. Then the dried electrodes (mass loadings of 3.5 and 3.2 mg cm^−2^ for the SG and SS samples, respectively) were pressed at 7 tons before being assembled in a CR2032 type coin cells 1 M NaClO_4_ (Organic Across, 99%) in EC:PC (Ethylene carbonate:Propylene carbonate, 1:1 by wt%) as electrolyte and Whatman GF/D borosilicate glass fibre as separator. The cell assembly was performed under argon atmosphere in a glovebox. The measurements were carried out using a MACCOR cycler operated in the working voltage window of 4.0–2.0 V vs. Na^+^/Na at C/10 and 1C, with C based on the theoretical capacity of O3-NaNMF (240.41 mA h g^−1^).

The operando X-ray diffraction pattern evolution of O3-NaNMF-SG at C/30 rate was collected every 45 min using a lab-scale Brüker D8 Advance X-ray diffractometer (CuKα) equipped with a LYNXEYE detector. The operando X-ray diffraction pattern evolution of O3-NaNMF-SS was recorded at C/10 rate using synchrotron light (*λ* = 0.8256 Å) at BL04-MSPD synchrotron beamline available at ALBA facilities (1 pattern every ∼5 min). All operando XRD experiments were performed using a homemade electrochemical cell equipped with a beryllium window as current collector, operating in reflection geometry in the case of laboratory measurements and in transmission geometry using a transparent plunger also equipped with a beryllium window in the case of synchrotron measurements. Cells were charged and discharged in the voltage window of 2–4 V using 1 M NaClO_4_ in EC:PC as electrolyte, Whatman GF/D borosilicate glass fibre as separator, and high purity sodium metal (Sigma-Aldrich) as an anode. The cell was galvanostatically cycled using a SP200 Biologic potentiostat.

### 2.7 Coupled PITT-EIS experiments

Potentiostatic Intermittent Titration Technique (PITT) was performed using a Bio-Logic VMP3 potentiostat, by applying successive constant potential steps of 25 mV, during which the current was allowed to relax down to 0.5 mA g^−1^, corresponding to C/500, with a duration limit of 20 h. At the end of each PITT relaxation step a potentiostatic EIS spectrum has been acquired with a frequency range 10 mHz–100 kHz.

## 3 Results and discussion

### 3.1 Structural and morphological characterizations

**FIGURE 1:**
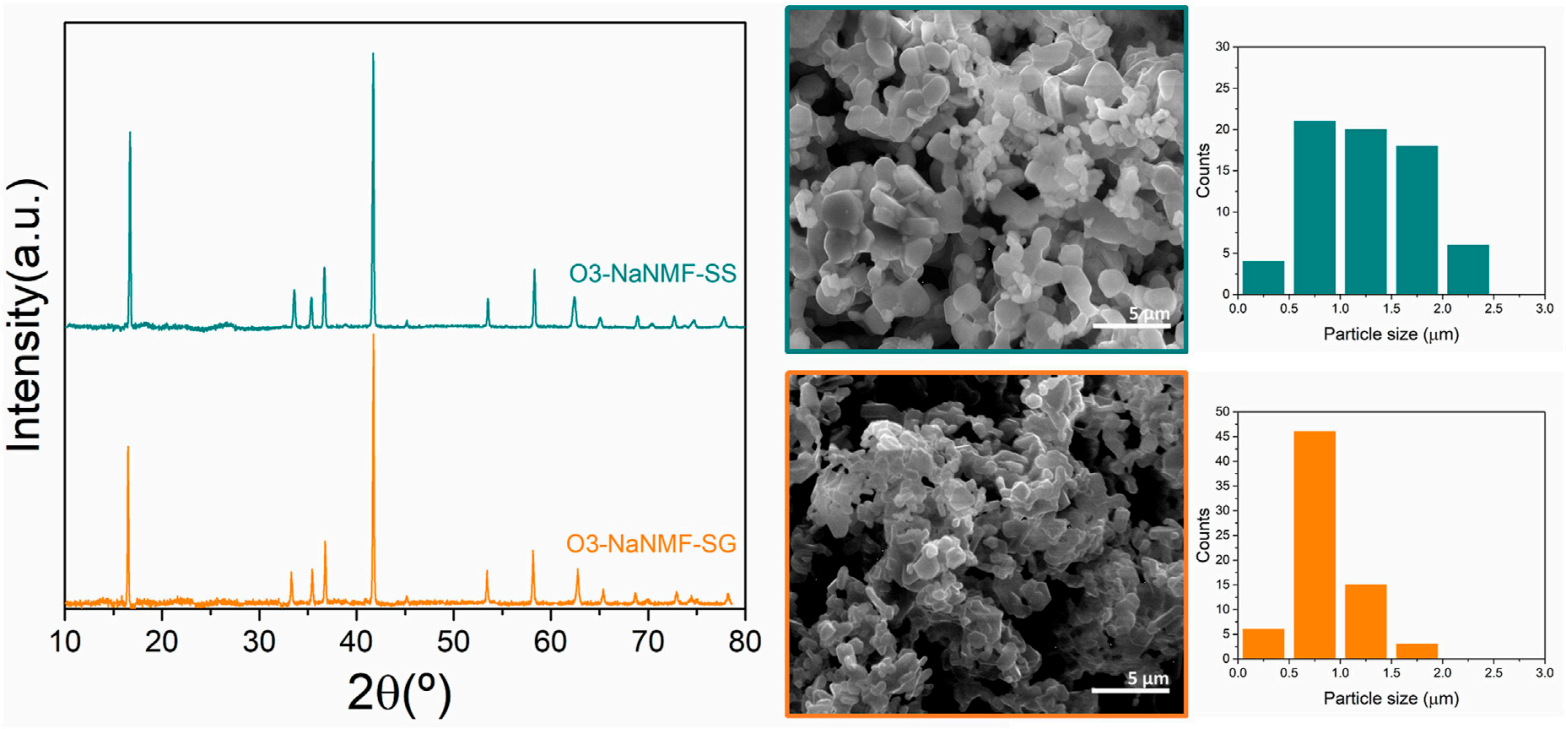
PXRD patterns and SEM images of O3-NaNMF synthesized *via* solid-sate (O3-NaMF-SS) and sol-gel (O3-NaMF-SG) routes.

**FIGURE 2:**
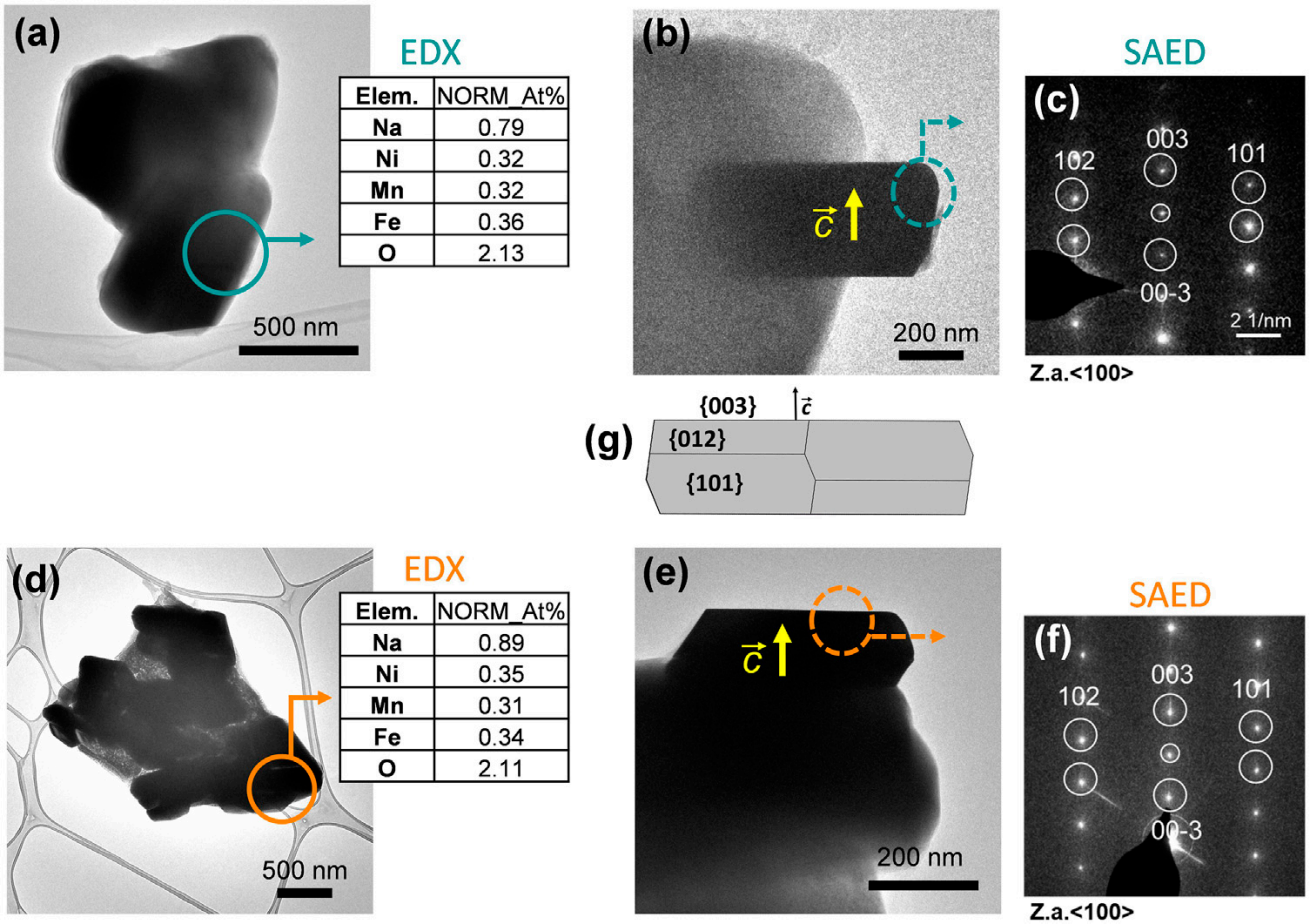
TEM images of the characteristic particles, EDX measurements and SAED patterns for **(A–C)** O3-NaNMF-SS and **(D–F)** O3-NaNMF-SG. Selected areas, where EDX measurements and electron diffraction patterns have been carried out, are highlighted. EDX quantifications are presented in atomic percentages and the values are normalized with respect to the values of Ni, Mn, Fe **(G)** The simulation of the habit of the particles.

The X-ray diffraction patterns of compounds synthesized *via* sol-gel and solid-state routes are shown in Figure 1. As can be seen, both materials are phase pure and exhibit good crystallinity, with strong peaks which may be assigned to the R-3m space group with the *a*-NaFeO_2_ structure (Gummow et al., 1992; Takeda et al., 1994). The cell parameters obtained *via* LeBail refinement (see Supplementary Figure S1) are summarized in Table 1, which shows good agreement with the literature (Kim et al., 2012). The average crystallite sizes determined from refinement for the O3-NaNMF-SS and O3-NaNMF-SG materials were found to be 140 and 55 nm, respectively (see Table 1). This is in agreement with the SEM images (Figure 1 **)**, which revealed that both materials present a good degree of homogeneity (with particles possessing size distributions in the range of 0.5–1.0 and 0.5–2.5 µm for sol-gel and solid-sate synthesis, respectively), but also showed that the solid-state particles are slightly larger with a more pronounced prismatic shape. The targeted O3-NaNMF compositions were verified by TEM-EDAX (see Figures 2A, D) measurement, which are consistent with the expected stoichiometry within the error of the determination, which agrees with ICP-OES measurements (Supplementary Table S1). Figures 2B, E shows TEM images of the characteristic particles and the electron diffraction patterns corresponding to [010] zone axis for each synthesized compound (Figures 2C, F). The tendency of the nanocrystals to grow into characteristic shapes was simulated using Shape software, based on Wulff plots (Dowty, 1995), and is also displayed in Figure 2G. According to the obtained morphology, the largest facets correspond to {003}, {102}, and {101} indices. Although the peaks (003)/(104) and (110)/(018) are very well resolved, indicating a high degree of crystallinity of the structure for both samples, the integrated intensity ratio I_(003)_/I_(104)_ is lower in the O3-NaNMF-SG sample (see Supplementary Figure S2). This indicates a higher degree of deviation from the ideal structure which may be attributed to transition metal (TM) migration in the sodium layers of O3-NaNMF-SG (i.e., transition metals and Na atoms exchanging their positions) (Gummow et al., 1992; Bréger et al., 2005; Li et al., 2016). This was substantiated by simulation of the XRD patterns with different degrees of TM migration (Supplementary Figure S3), where the % of TM migration was found to be ca. 10% for O3-NNMF-SG and ca. 0%–2.5% for O3-NNMF-SS.

**TABLE 1:** Unit Cell Parameters and crystallite size of synthesis compounds obtained by Le Bail refinements.

Sample	Unit cell parameters (Å)		Crystallite size (nm)
	a = b	c	
O3-NaNMF-SS	2.9803 (1)	16.0717 (9)	140 ± 41
O3-NaNMF-SG	2.9656 (2)	16.1204 (2)	55 ± 15

### 3.2 Electrochemical characterization

**FIGURE 3:**
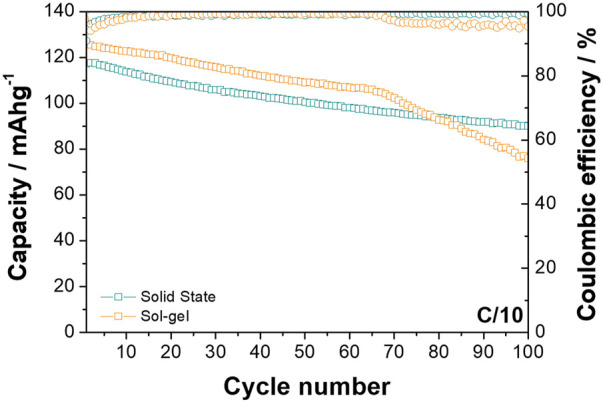
Cyclability plots of O3-NaNMFO-SS (dark turquoise) and O3-NaNMFO-SG (orange) at C/10.

**FIGURE 4:**
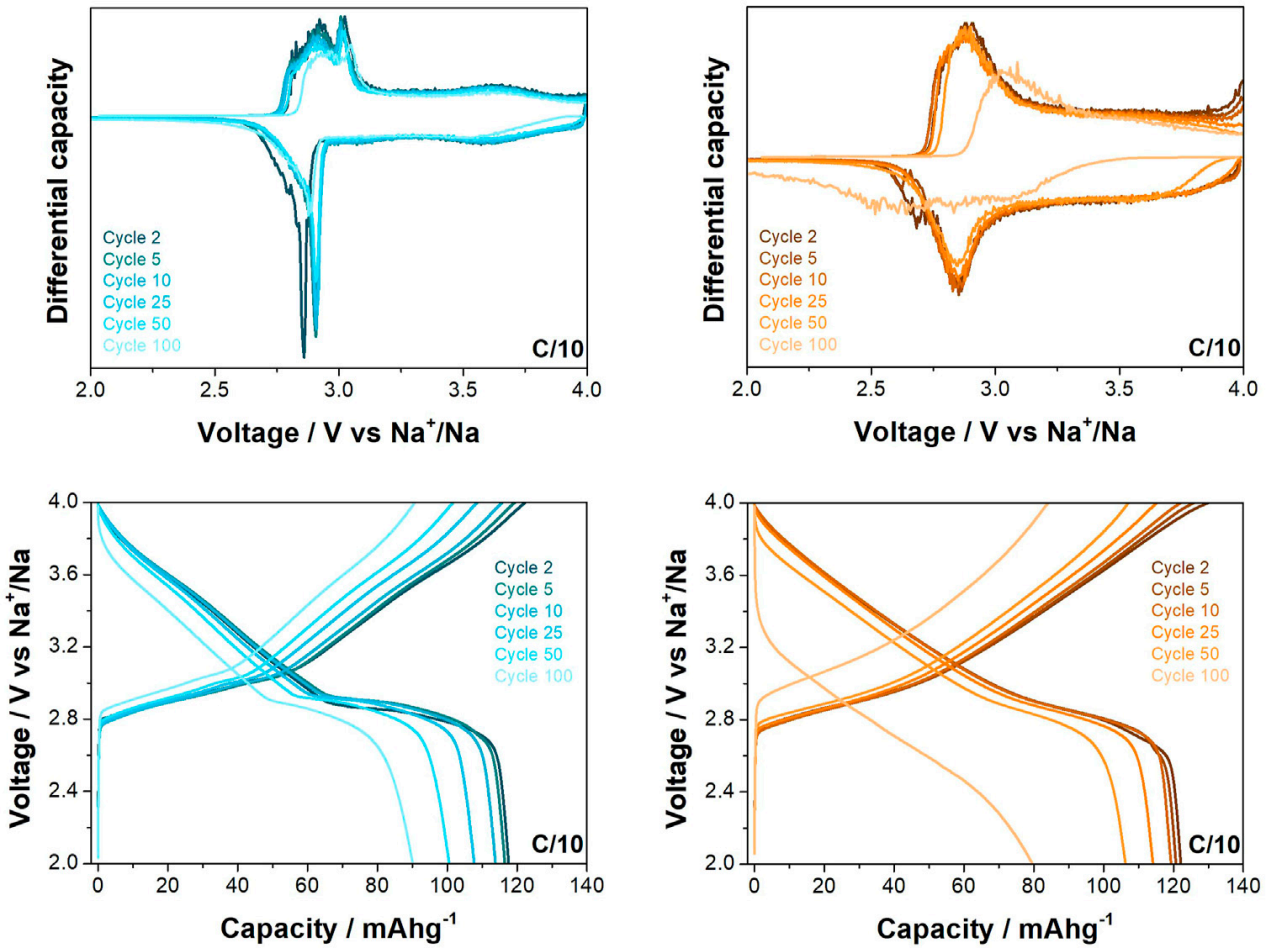
Differential capacity plots and load curves for O3-NaNMFO-SS (dark turquoise) and O3-NaNMFO-SG (orange) at C/10.

The materials were cycled between 2.0 and 4.0 V vs. Na^+^/Na at C/10 (where C is taken as 240.41 mA h g^−1^, based on the theoretical capacity of the materials) for 100 cycles (see Figure 3), with the O3-NaNMF-SG sample thereafter exhibiting a noticeable decrease in stability while the O3-NaNMF-SS sample maintained this good cyclability over the full 100 cycles. From this it can be seen that the O3-NaNMF-SG offers a slightly higher capacity than the O3-NaNMF-SS (see Table 2). Closer examination of the differential capacity plots and load curves for the O3-NaNMF-SG and O3-NaNMF-SS materials at this rate (see Figure 4) shows that both materials are broadly similar and exhibit redox activity in keeping with that previously reported—namely, a notable peak in the 2.7–3.1 V vs. Na^+^/Na region (which has previously been assigned to the Ni component and the reversible phase transition between the O3 and P3 phases) and subsequently a much broader peak at higher voltages (which has previously predominantly been attributed to additional Ni^3+/4+^ and/or Fe^3+/4+^ redox activity, as well as homogenous higher-voltage phase processes) (Xie et al., 2016; Zhang et al., 2017; Sun et al., 2018; Xie et al., 2018; Jeong et al., 2019; Jung et al., 2020). However, the plateau at 2.7 V vs. Na^+^/Na is more pronounced for O3-NaNMF-SS, leading to sharper peak in the derivative curve - suggesting the synthesis route had an effect on the Na insertion-extraction process. As can be seen, both materials exhibited good stability over the 50 cycles, with relatively little reduction in redox activity (which is proportional to the relatively little reduction in capacity on cycling observed in the cyclability plot, Figure 3). Examination of the dQ/dV plots and the voltage profile (Supplementary Figure S4) reveals that while the difference between the potentials at which the charge and discharge peaks occur is relatively stable for the O3-NaNMF-SG material during the first 50 cycles, the O3-NaNMF-SS material appears to exhibit an initially greater difference which rapidly decreases by cycle 5 to be less than that of the O3-NaNMF-SG and thereafter remains more stable by cycle 100. This would seem to suggest that, overall, the stability and the Coulombic efficiency of the O3-NaNMF-SS material is greater than that of the O3-NaNMF-SG, implying that the choice of synthetic route has significant consequences with respect to electrochemical behaviour, especially during the later cycles. This phenomenon may tentatively be attributed to the higher degree of TM migration observed in the initial structure of O3-NaNMF-SG using XRD (see previous section), particularly as the presence of TM in the Na layers is a known source of capacity loss in transition metal layered oxides (Gummow et al., 1992; Bréger et al., 2005; Li et al., 2016). Furthermore, in the dQ/dV plot for the O3-NaNMF-SG material, it can be seen that there is activity in the region near 4.0 V vs. Na^+^/Na which is not apparent in O3-NaNMF-SS (Figure 4).

**TABLE 2:** Tabulated electrochemical performance at C/10.

Material	Discharge capacity at cycle/mAhg^-1^					Cycle 100 capacity retained/%	Energy density/Wh kg^-1^	Average Voltage/V
	2	5	10	50	100			
O3-NaNMF-SS	117.60	116.48	113.81	100.53	90.11	77	367.08	3.12
O3-NaNMF-SG	125.50	124.06	122.77	109.05	76.04	61	393.44	3.13

**FIGURE 5:**
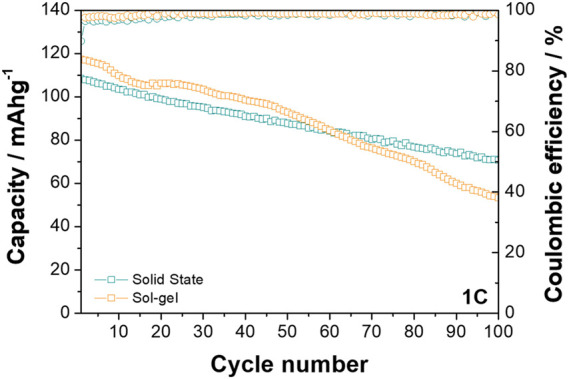
Cyclability plots of O3-NaNMFO-SS (dark turquoise) and O3-NaNMFO-SG (orange) at 1C.

**FIGURE 6:**
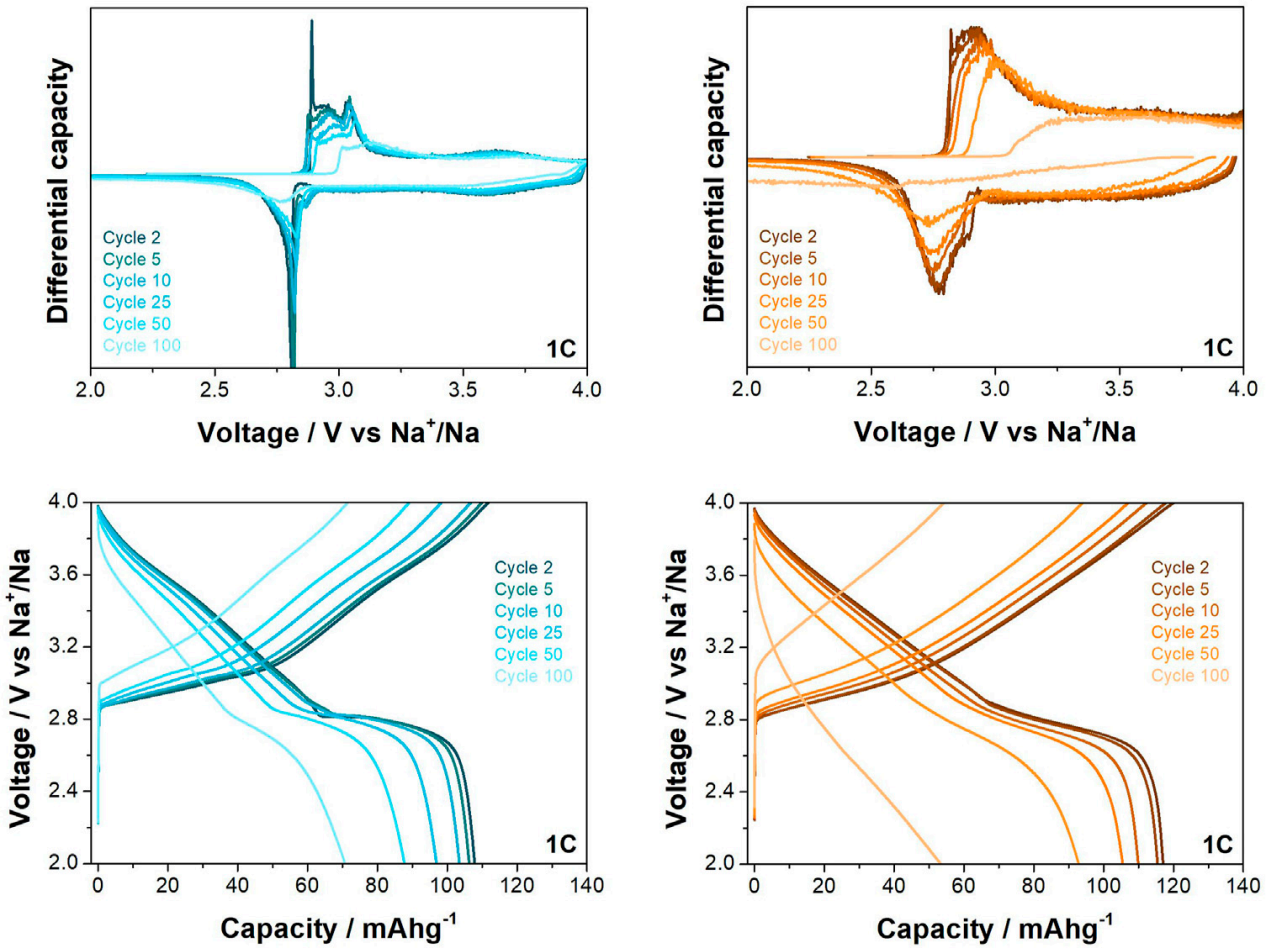
Differential capacity plots and load curves for O3-NaNMFO-SS (dark turquoise) and O3-NaNMFO-SG (orange) at 1C.

Differences between the behaviour of the two materials is also observed at the higher rate of 1C when cycled between 2.0 and 4.0 V vs. Na^+^/Na (see Figure 5). While the O3-NaNMF-SG again displayed a higher initial capacity, the solid state showed superior cyclability over the 100 cycles (see Table 3 **)**. An examination of the differential capacity plots and load curves was undertaken (see Figure 6) and the O3-NaNMF-SS material exhibits notably greater stability of both electrochemical peaks, with the decrease in capacity arising predominantly from the loss of redox activity in the 2.7–3.1 V vs. Na^+^/Na region.

**TABLE 3:** Tabulated electrochemical performance at 1C.

Material	Discharge capacity at cycle/mAhg^-1^					Cycle 100 capacity retained/%	Energy density/Wh kg^-1^	Average voltage/V
	2	10	25	50	100			
O3-NaNMF-SS	107.86	103.49	96.92	87.77	70.76	66	336.19	3.12
O3-NaNMF-SG	117.04	108.69	105.46	92.86	53.40	46	363.23	3.10

Finally, it should be noted that both O3-NaNMF samples offer excellent performance compared to those previously reported in the literature—able to provide a capacity similar or superior to other well-optimised materials at the same rate, they also offer cyclability comparable or superior to materials cycled at much lower rates (SupplementaryTable S2).

In order to further investigate these differences in electrochemical behaviour, operando XRD was carried out on both materials to elucidate structural changes during charge and discharge.

### 3.3 Operando XRD

**FIGURE 7:**
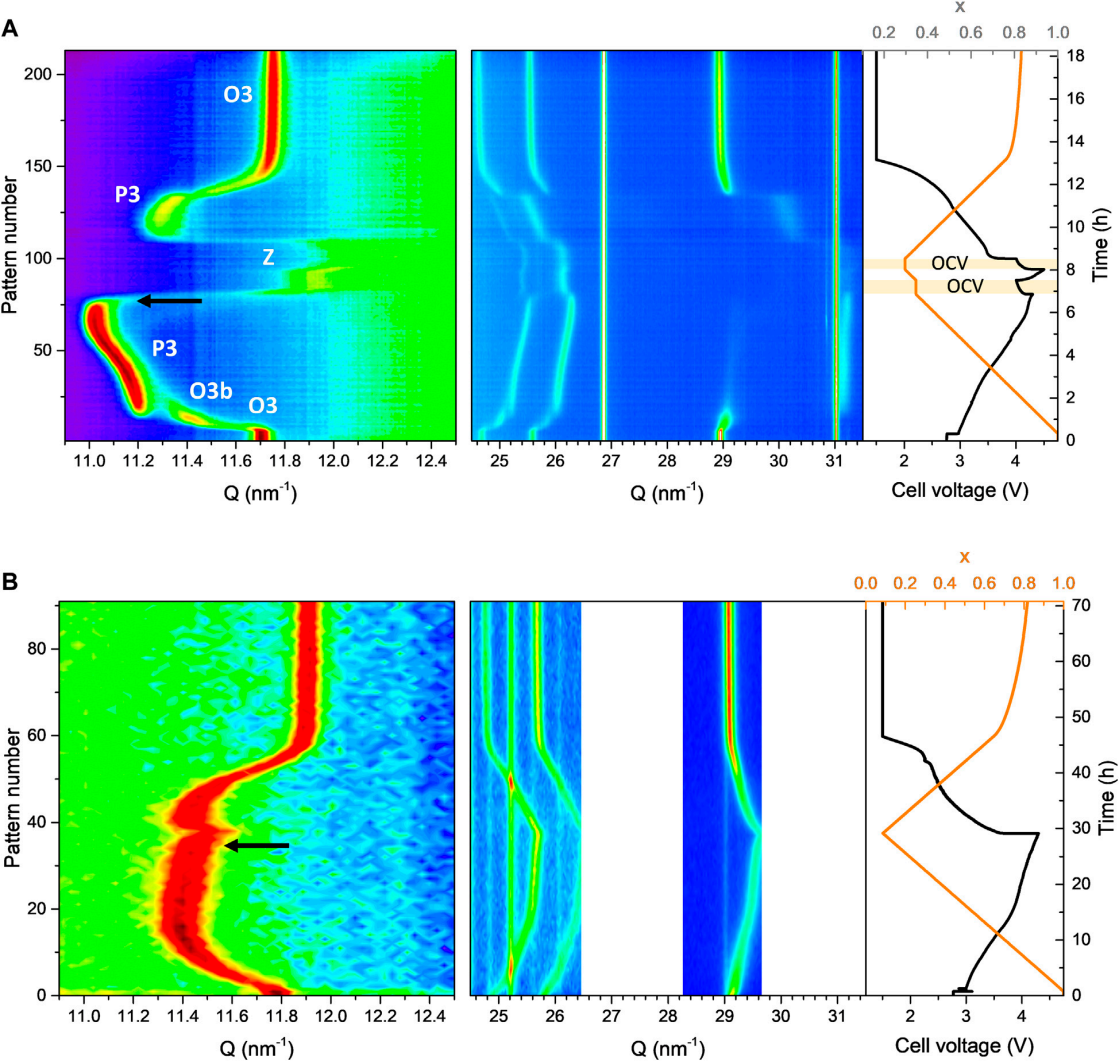
Operando XRD taken during the first cycle charge of **(A)** O3-NaNMFO-SS and **(B)** O3-NaNMFO-SG. The black arrows indicate the moment when the (003) peak starts to shift to larger angle.

The O3-NaNMF-SS sample shows the expected structural evolution upon Na^+^ extraction (see Figure 7A), with the initial O3 phase transforming fully into a P3 phase. The transition from O to P stacking is demonstrated by the disappearance of the (104) reflection concomitant with the (105) reflection appearing near 1.5 h (c.a. x = 0.85), as previously reported et al., 2016). Interestingly, the O3 to P3 transition occurs through an intermediate O3b phase. This O3b phase seems similar to the initial O3 phase, albeit with shifted peaks indicating difference in cell parameters (and probably also Na content). There is no peak splitting, peak extinction or new peak appearing, which indicates this phase has same space group than the initial O3 phase (e.g., without monoclinic distortion). Such intermediate O3b phase has not been previously reported for this compound, possibly because the time resolution of previous experiments did not allow its observation. After 6.5 h (x <0.4), the P3 phase vanishes, transforming into a new phase with lower interlayer distance indicated by the strong high angle shift of the (00l) reflections. According to previous studies, this phase is an OP or “Z” phase (depending if ordered or disordered) made of O and P alternate stacking (Xie, et al., 2016). This phase transition must be avoided for good cycling, as the strong shrinking of the interlayer distance makes it prone to structural instabilities such as TM migration and O redox (Yabuuchi et al., 2012; Talaie et al., 2015; Rong et al., 2019). For this reason, such materials are usually cycled in the range of 2–4.3 V vs. Na^+^/Na, ensuring the P3-OP/Z transition is not triggered.

Surprisingly, the structural evolution upon Na^+^ extraction of the O3-NaNMF-SG sample exhibits strong differences compared to that of the O3-NaNMF-SS sample (see Figure 7B). Here no phase transition is observed, the structure remaining O3 during the whole Na^+^ extraction down to x_Na_ = 0.1. This is confirmed by the persistence of the (104) reflection. A possible explanation is that the higher degree of TM migration present in the as-prepared O3-NaNMF-SG sample would forbid layer gliding, which is required for the O3-P3 transition to occur (Vassilaras et al., 2017; Xiao et al., 2021). Interestingly, around half charge (4 V vs. Na^+^/Na and xNa ∼ 0.5) a change in the trend of the (003) reflection is observed for the O3-NaNMF-SG sample: while it is shifted toward lower angle (indicating interlayer distance expansion) at the beginning of charge, it shifts toward larger angle above xNa ∼ 0.5 (indicating interlayer distance contraction). The interlayer distance in sodium transition metal layered oxides is known to be highly dependent on sodium content, and commonly assumed to result from the balance between O-O electrostatic repulsion and Van der Waals attraction forces (Mortemard de Boisse et al., 2019). At high sodium content, the Na is screening the O-O repulsion, so removal of sodium induces increase of interlayer distance. However, below a certain Na concentration, Van der Waals forces between the TM-O layers start to enter into play, shrinking the interlayer distance. This is when the layered structure starts to lose its stability and structural degradation phenomena such as TM migration into the Na layers (Silván et al., 2018), or O redox activity (Li et al., 2018; Susanto et al., 2019), may enter into play. This is further confirmed, for when both materials were charged beyond this inflection of the interlayer distance, as indicated by the black arrow (see Figure 7), in both cases there is a notable loss of reversibility upon discharge.

It is reasonable to consider the beginning of interlayer distance shrinking as the onset of such degradation, implying it occurs at x = 0.4 (4.2 V) for O3-NaNMF-SS sample, while for O3-NaNMF-SG sample it occurs at x = 0.6 (3.8 V). This means that the O3-NaNMF-SS sample can sustain reversible cycling up to 4.2 V, while for the O3-NaNMF-SG sample charge voltage would need to be limited to 3.8 V to ensure good cycling. This offers an explanation for the difference in capacity fading when cycled in the 2.0–4.0 V vs. Na^+^/Na window (see Figures 4, 6), where the fading of the O3-NaNMF-SG material is greater than that of the O3-NaNMF-SS material.

These expectations correspond well with the experimental data, offering a good explanation for the observed differences in performance. It might seem counter intuitive that the sample showing less phase transition (the O3-NaNMF-SG sample) to exhibit poorer cyclability than the sample showing clear phase transition (O3-NaNMF-SS), because it is well known that such phase transitions, occurring through gliding, tend to generate overpolarization (Sun et al., 2019). However, here it is the stability of the phase present at the end of charge that has the most significant effect on cyclability, and the P3 phase of O3-NMF-SS allows more Na extraction before the interlayer distance collapses than the O3 phase of O3-NMF-SG.

### 3.4 PITT-EIS studies

**FIGURE 8:**
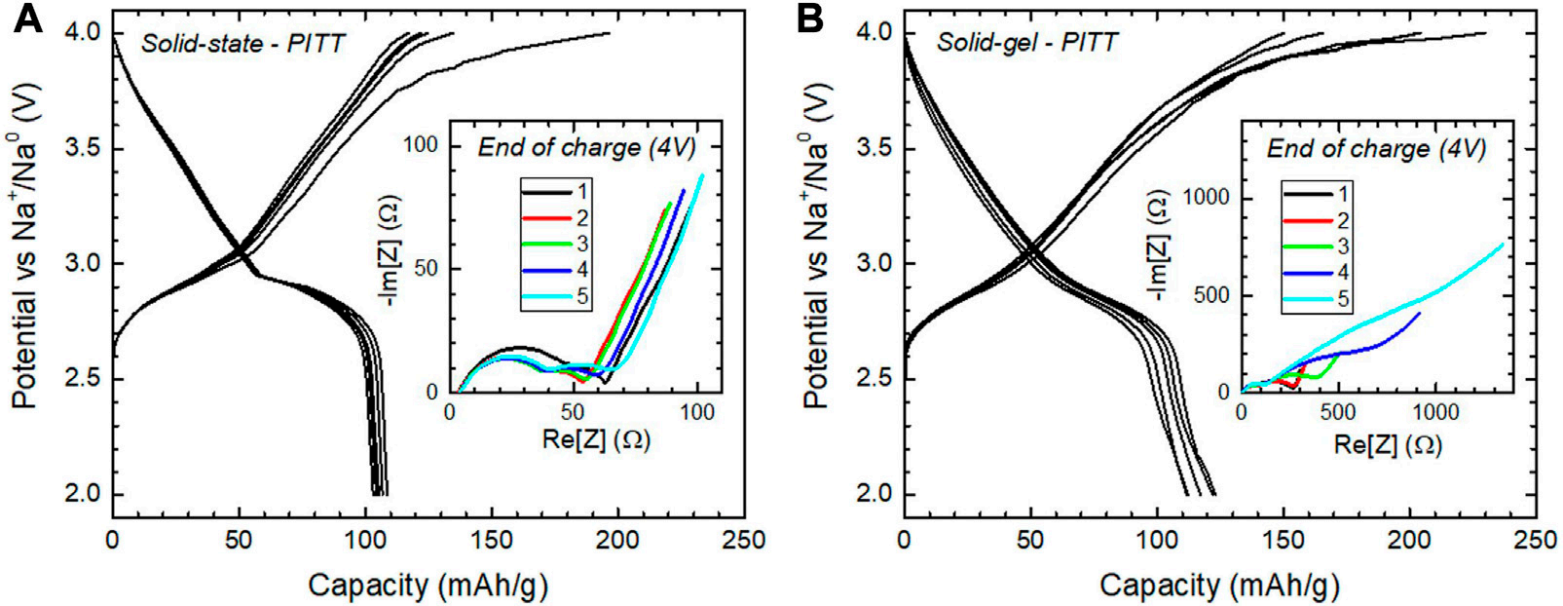
Results from the coupled PITT-EIS experiment of **(A)** O3-NaNMF-SS and **(B)** O3-NaNMF-SG. Main panels: PITT curve for five cycles. Insets: EIS pattern at the end of each PITT charge (4 V).

The voltage composition profile at equilibrium shows a side reaction at the end of the first charge on both samples denoted by an extended plateau, see Figure 8 (main panels), albeit more pronounced for O3-NaNMF-SG. Interestingly, while for O3-NaNMF-SS the next cycles no longer present this side reaction, it is sustained upon cycling for O3-NaNMF-SG. As a consequence, discharge capacity is more stable for O3-NaNMF-SS than O3-NaNMF-SG. Nyquist plots of EIS recorded at the end of charge show marginal evolution upon cycling for O3-NaNMF-SS (inset of Figure 8A), while huge increase of impedance is observed for O3-NaNMF-SG upon cycling (inset of Figure 8B).

This increase of impedance relies essentially on the second semi-circle, which is usually ascribed to charge transfer, while the semi-circle at higher frequency is usually ascribed to interface processes. This means the degradation of the electrochemical performance relates to a charge transfer that is less and less facile. A similar effect was previously reported for NaFeO_2_, and ascribed to the effect of TM migration into the Na layers of the O3 phase (Silván et al., 2018). Although the texture and chemistry of the surface might also have an influence, it seems reasonable to ascribe this increase of charge transfer resistance to the nature of the main phase, being O3-type in O3-NMF-SG and P3-type in O3-NMF-SG.

### 3.5 Post cycling studies

**FIGURE 9:**
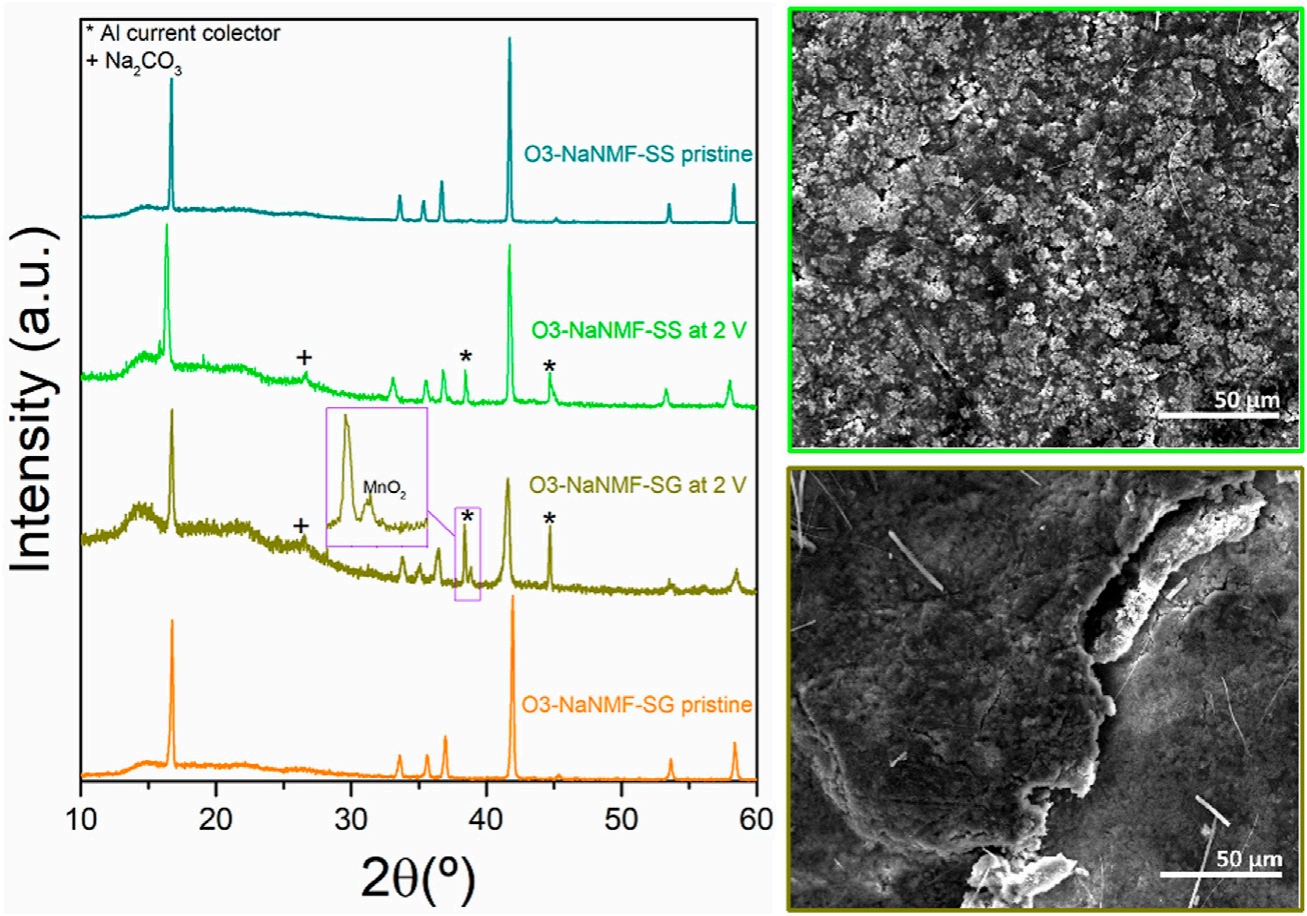
*Ex-situ* XRD patterns of O3-NaNMF-SS of O3-NaNMF-SG pristine electrodes as well as the cycled ones after 100 cycles at 2 V and their corresponding SEM images.

In order to get further insight into the degradation mechanism occurring over long-term cycling, post cycling studies were carried out. The structural stability of both O3-NaNFM-SS and O3-NaNFM-SG after 100 cycles was evaluated by means of *ex-situ* XRD measurements of both electrodes in discharge state at 2 V vs. Na^+^/Na. From this (see Figure 9) it can be observed that after 100 cycles both the O3-NaNMF-SS and the O3-NaNMF-SG samples maintained an O3 crystalline structure similar to the pristine electrodes. This indicates a relatively high degree of reversibility of both cathodes after 100 cycles. However, the faster capacity decay of the O3-NaNMF-SG sample (comparative to the O3-NaNMF-SS) could be related to the observations that after 100 cycles it possesses a lower degree of crystallinity (broadening of the XRD peaks) and has formed some MnO_2_ (as indicated by the presence of a small extra peak at 2θ ≈ 39°). Significantly, no identifiable difference between the O3-NaNMF-SG and O3-NaNMF-SS Na-metal counter electrodes was observed, which suggests that the dominating factor for performance differences indeed results from the cathode.

The lower crystallinity of the O3-NaNMF-SG sample may be attributed to a larger degree of TM migration to the Na layer. This is in keeping with the previous results, as in the cycling window 2.0–4.0 V vs. Na^+^/Na, the O3-NaNMF-SS sample is expected to be P3 at the end of charge, while the O3-NaNMF-SG sample is expected to remain O all along the charge. It has previously been reported that the P phase is unfavourable for TM migration while the O phase is not (Silván et al., 2018). Consequently, it is reasonable to expect the O3-NaNMF-SG phase, with the defect induced suppression of P phase transformation, will be more susceptible to TM migration than the O3-NaNMF-SS material. Meanwhile, the formation of MnO_2_ may be tentatively attributed to dissolution of Mn from cathode/electrolyte interface, resulting from the disproportionation reaction (2Mn^3+^ → Mn^2+^ + Mn^4+^) and decreased structural stability of O3-NaNMF-SG (Xia et al., 1997; Lu et al., 2014; Guo et al., 2017). In order to more conclusively validate these hypotheses, future analyses (such as EXAFS, XANES, NMR, etc.) could be carried out, so as to elucidate any effect of other factors (including those such as disorder or compositional homogeneity).

In this way, these results support the previous observations. The O3-NaNMF-SG material appears to be more prone to TM migration [and thus loss of crystallinity and decreased (de) intercalation ability] than the O3-NaNMF-SS, due to the continual presence of the O phase (which is more susceptible to TM migration) during cycling, which in turn results from suppressing the structural evolution to the P phase upon charge.

## 4 Conclusion

O3-NaNMF samples were synthesized *via* two different techniques (sol-gel and solid state), both of which are fast, facile, sustainable lab-scale routes able to support future sodium layered oxide development. However, while both materials offer excellent performance, key differences in performance and behaviour were also observed, especially a faster capacity fading for the O3-NaNMF-SG sample after 60 cycles. This, therefore, has significant implications on investigations into such materials with respect to suitability for high-power or fast-charging applications.

In depth characterisation of the structural changes during cycling was carried out using in operando XRD. It has been shown that while the expected O3-P3 transition was observed for the O3-NaNMF-SS sample, it did not occur in the O3-NaNMF-SG sample. This may be attributed to the presence of TM migration in the SG sample after synthesis preventing the layer gliding required for the O3-P3 transition to occur. While typically decreased phase changes during cycling may be thought of as beneficial to performance, in fact we see the opposite. This may be explained by the differences in susceptibility to TM migration of the phase at end of charge, which is less favourable in P structure. Consequently, it may be expected that the O3-NaNMF-SG will be more prone to TM migration (and thus decreased crystallinity and performance) than the O3-NaNMF-SS, which would explain the differences in observed cycling stability. Thus, TM migrated to the Na layer have two distinct effects in the O3-NaNMF-SG sample. On one hand, TM migration in as prepared material forbids the expected O3-P3 transition upon cycling, which tends to smooth-out slightly the voltage-composition profile compared to O3-NaNMF-SS sample. This absence of gliding-related phase transition would be beneficial to cycle life. On the other hand, as a consequence of the latter, the material remains O3 (instead of transforming to P3) at end of charge (when approaching 4 V vs. Na^+^/Na), in which case further TM migration to the Na layer occurs—eventually leading to a degradation of the electrochemical performance.

Finally, it is worth highlighting these results demonstrate that selection of synthetic route—a frequently neglected descriptor—can have a substantial impact on the physico- and electrochemical properties of ostensibly the same material. Moreover, the techniques outlined here represent an important toolkit for materials development, which can help understand what phenomena are responsible for differences in performance. Consequently, this work offers a good foundation for approaches in analysing sodium-based layered oxide materials—facilitating the comparison between experimentally derived performances by helping to understand the root causes, a key issue when developing novel materials.

## Data Availability

The raw data supporting the conclusion of this article will be made available by the authors, without undue reservation.
