# Supplementary material for: Investigating the effect of synthesis selection on O3-sodium layered oxide structural changes and electrochemical properties
Source: Front Chem. 2023 Apr 6;11:1151656. doi: 10.3389/fchem.2023.1151656 (PMC10117976; doi:10.3389/fchem.2023.1151656)
Supplement: Supplementary file 1 [file DataSheet1.pdf]

Supplementary Material

Investigating the effect of synthesis selection on O3-sodium layered oxide structural changes and electrochemical properties

L. Acebo[1,2], N. E. Drewett[1*], D. Saurel[1], F. Bonilla[1], T. Rojo[2] and M. Galceran[1*]

[1] Center for Cooperative Research on Alternative Energies (CIC EnergiGUNE), Basque Research and Technology Alliance (BRTA), Parque Tecnológico de Alava, Albert Einstein 48, 01510, Vitoria-Gasteiz (Spain)

[2] Departamento de Química Orgánica e Inorgánica, Universidad del País Vasco UPV/EHU, P.O. Box 644, 48080 Bilbao (Spain)

*** Correspondence:** Corresponding Author: ndrewett@cicenergigune.com; mgalceran@cicenergigune.com

Table S1. Comparative table summarizing the electrochemical performance of the reported O3-NaNMF with our O3-NaNMF-SG and O3-NaNMF-SS studied in this work.

	ICP Concentration (ppm)/Experimental molar ratio		
**Sample**	**Ni**	**Mn**	**Fe**
**O3-NNMF-SS**	0.3401	0.3187	0.3412
**O3-NNMF-SG**	0.3429	0.3281	0.329

Note: Concentration (in ppm) of the metallic elements detected in the samples by ICP-OES measurement together with the atomic ratio of metallic elements in the samples, determined by setting [TM] = 1, adapted from (Gonzalo *et al.*, 2018). *Note errors on the reported values are 5%.*

Table S2. Comparative table summarizing the electrochemical performance of the reported O3-NaNMF with our O3-NaNMF-SG and O3-NaNMF-SS studied in this work.

**Reference**	**Rate / mA g^{-1}**	**Capacity / mAh g^{-1}**	**Capacity retention over 50 cycles**	**Synthetic route**
**This Work O3-NaNMF-SG**	240	117	79%	Sol-gel (resorcinol-formaldehyde)
**This Work O3-NaNMF-SS**	240	108	81%	Solid-state (mixed carbonates)
**O3-NaNMF** (Zhang *et al.*, 2017)	150	118	81%	Solid-state (mixed oxides)
**O3-NaNMF** (Wang *et al.*, 2016)	130	123	90%	Coprecipitation
**O3-NaNMF** (Jung *et al.*, 2020)	120	113	42%	Coprecipitation
	240	83	-	
**O3-NaNMF** (Sun *et al.*, 2018)	130	122	90%	Coprecipitation

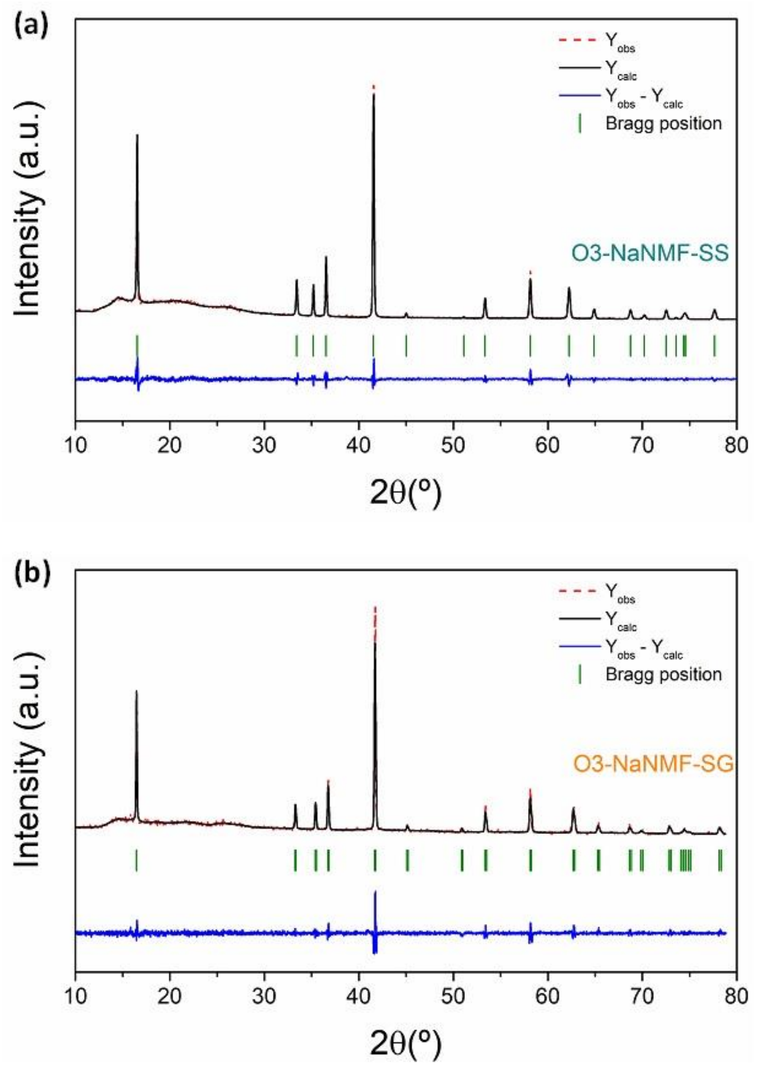

Figure S1. Le Bail Refinements of the O3-NNMF obtained by (a) solid-state and (b) sol-gel synthesis routes.

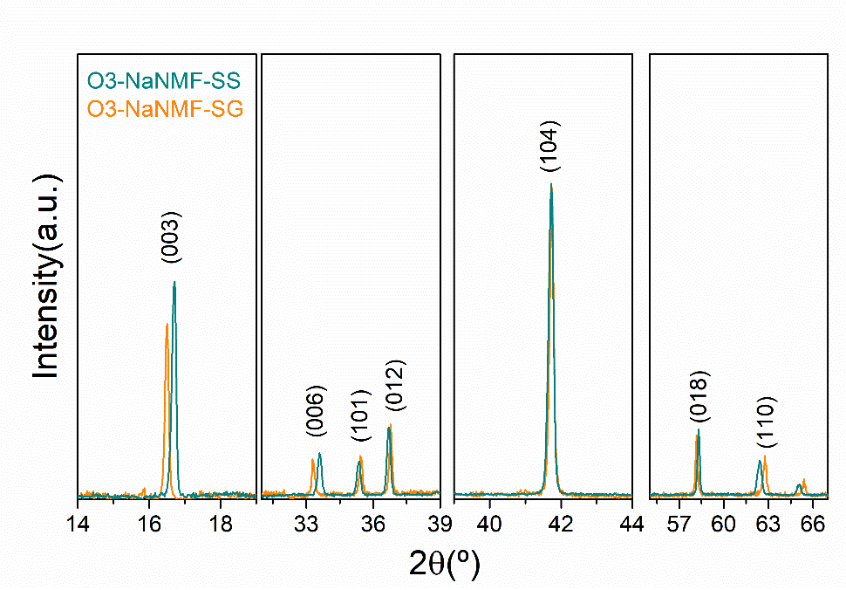

Figure S2. Comparison of the ratio of the intensities of the (003)/(104), (006)/(101) and (018/110)

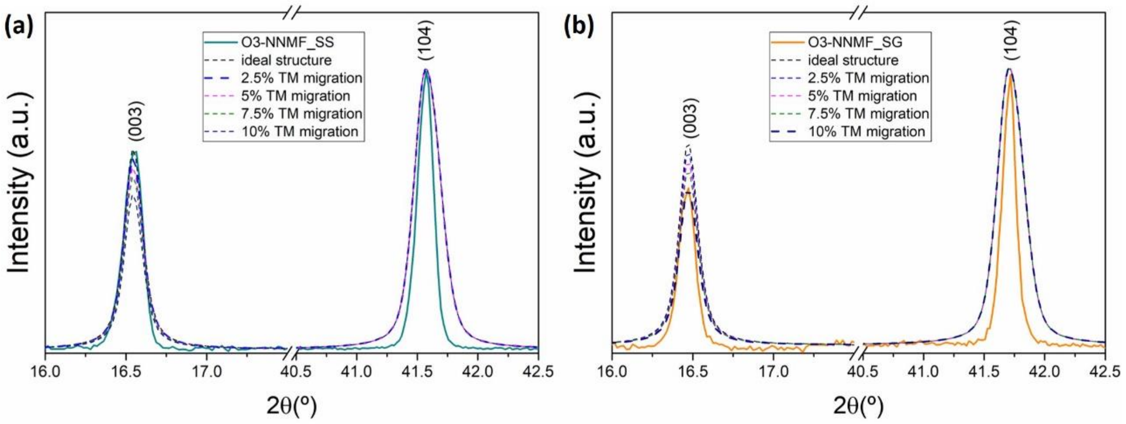

Figure S3. Simulation of diffractions (003), (104) for O3-NNMF-SG and O3-NNMF-SS as a function of the % of the TM migration to sodium layer

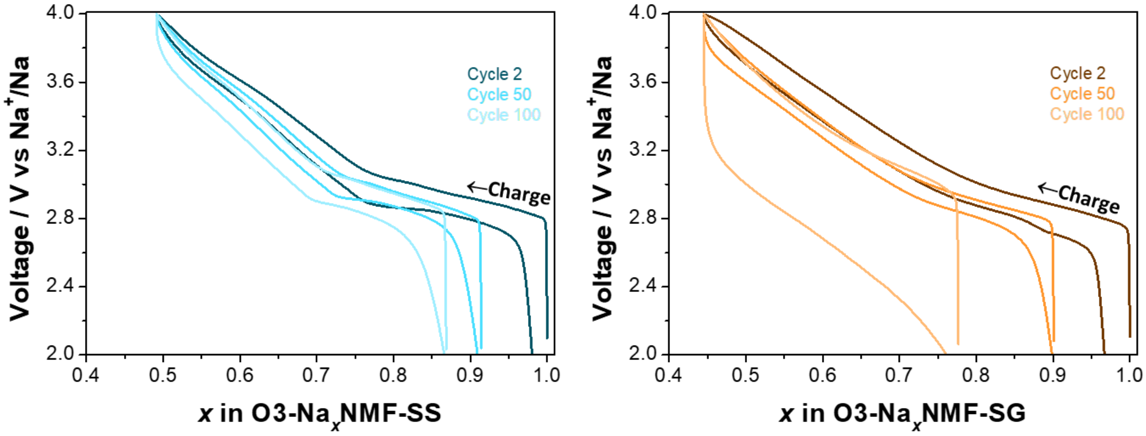

Figure S4. Voltage vs bulk stoichiometry plots for both synthesized materials.

References

Gonzalo, E. *et al.* (2018) ‘P2 manganese rich sodium layered oxides: Rational stoichiometries for enhanced performance’, *Journal of Power Sources*, 401, pp. 117–125. doi: 10.1016/j.jpowsour.2018.08.068.

Jung, K. N. *et al.* (2020) ‘Mg-doped Na[Ni1/3Fe1/3Mn1/3]O2 with enhanced cycle stability as a cathode material for sodium-ion batteries’, *Solid State Sciences*. Elsevier Masson SAS, 106(March), p. 106334. doi: 10.1016/j.solidstatesciences.2020.106334.

Sun, L. *et al.* (2018) ‘Insight into Ca-Substitution Effects on O3-Type NaNi1/3Fe1/3Mn1/3O2 Cathode Materials for Sodium-Ion Batteries Application’, *Small*, 14(21), pp. 1–7. doi: 10.1002/smll.201704523.

Wang, H. *et al.* (2016) ‘Large-Scale Synthesis of NaNi 1/3 Fe 1/3 Mn 1/3 O 2 as High Performance Cathode Materials for Sodium Ion Batteries’, *Journal of The Electrochemical Society*, 163(3), pp. A565–A570. doi: 10.1149/2.0011605jes.

Zhang, Q. *et al.* (2017) ‘F-doped O3-NaNi1/3Fe1/3Mn1/3O2 as high-performance cathode materials for sodium-ion batteries’, *Science China Materials*, 60(7), pp. 629–636. doi: 10.1007/s40843-017-9045-9.
